# Introducing Sharing Information to Stop Mass Shootings (SISMS): A Short Report on a Behavioral and Event-Level Dataset for the Study of Mass Public Shootings

**DOI:** 10.1007/s12103-026-09921-3

**Published:** 2026-06-29

**Authors:** Jaclyn Schildkraut, Emily A. Greene-Colozzi, M. Hunter Martaindale

**Affiliations:** 1 Rockefeller Institute of Government, 411 State Street, Albany, NY 12203 USA; 2 University of Massachusetts Lowell, Lowell, USA; 3 ALERRT, Texas State University, San Marcos, USA

**Keywords:** Mass public shootings, Targeted violence, Pre-attack behaviors, Leakage, Violence prevention

## Abstract

Mass public shooting research has been hindered by inconsistencies in case definitions, limited behavioral data, and variation in methodological transparency across existing datasets. This short report introduces Sharing Information to Stop Mass Shootings (SISMS), a behavioral and event-level dataset designed to support research on mass public shootings in the United States. SISMS currently includes 171 incidents and 175 perpetrators occurring between 1999 and 2024 for which pre-attack warning behaviors or leakage were confirmed through available source materials and incorporates data derived from public records, official documents, and triangulated open-source reporting. In addition to demographic and incident characteristics, the dataset includes information related to grievances, concerning behaviors, planning and preparation, leakage, and other pre-attack indicators associated with pathways to intended violence. This report describes the dataset structure, inclusion criteria, source collection procedures, and presence-based coding methodology. Potential applications for research and violence prevention also are discussed.

Mass public shootings remain an area of significant concern for researchers, policymakers, and practitioners, particularly given their considerable social, political, and public health impacts. In response, scholarly interest in mass shootings has expanded substantially over the last decade (Kim et al., 2021). Despite this growth, the data infrastructure supporting research on mass shootings remains comparatively underdeveloped. Existing databases often vary considerably in their definitional parameters, inclusion criteria, coding procedures, and methodological transparency (Booty et al., 2019; Schildkraut & Lankford, 2024). Such inconsistencies complicate cross-study comparisons and contribute to challenges in building cumulative knowledge on the phenomenon.

At the same time, many publicly available mass shooting datasets rely heavily or exclusively on media sources as the foundation for case identification and data collection. Although open-source reporting has enabled researchers to systematically study a relatively rare form of violence, media-based data collection presents important methodological limitations related to coverage disparities, incomplete information, evolving investigative details, and variability in reporting practices across outlets (Chermak et al., 2025; Huff-Corzine et al., 2014; Schildkraut et al., 2018). Further, many existing datasets contain limited information related to perpetrators’ pre-attack behaviors, grievances, warning signs, communications, and other behavioral indicators associated with pathways to intended violence.

To address these gaps, this short report introduces Sharing Information to Stop Mass Shootings (SISMS), a behavioral and event-level dataset designed to support research on mass public shootings in the United States. SISMS incorporates linked event- and perpetrator-level data derived from official records, court documents, investigative materials, summary reports, and triangulated media sources. In addition to demographic and incident characteristics, the dataset includes extensive information related to grievances, concerning behaviors, planning and preparation, leakage (i.e., the communication of intent to do harm to a third party; see Meloy & O’Toole, 2011), interpersonal stressors, and other pre-attack indicators. The purpose of this report is to describe the structure, development, and coding procedures of the SISMS dataset, with particular attention to its multilevel design, source collection framework, and presence-based coding methodology.

## Dataset Overview

SISMS is a multilevel dataset designed to support research on mass public shootings in the United States. The current release includes 171 incidents and 175 perpetrators occurring between 1999 and 2024 and represents Phase 1 of a larger dataset development project focused on incidents in which pre-attack warning behaviors or leakage were documented through available source materials (see also Greene-Colozzi, 2022). Additional mass public shooting incidents meeting inclusion criteria through 2026 (see Schildkraut et al., 2026) currently are undergoing coding and verification procedures and will be incorporated as part of a planned Phase 2 release in 2027.

Consistent with prior work (Schildkraut & Elsass, 2016), mass public shootings are defined as incidents of targeted violence involving one or more shooters at one or more public or populated locations in which multiple victims are injured and/or killed. Victims and locations are selected either at random or for their symbolic value, and incidents related primarily to gang violence or coordinated group-based terrorism are excluded. Prior clarifications of this definition further note that ideologically motivated lone actors acting independent of formal terrorist organizations remain eligible for inclusion (Schildkraut, 2021). Accordingly, case inclusion was determined based on the broader context and circumstances of the attack rather than a standalone numerical casualty threshold. Cases were identified through existing databases, media searches, and supplementary open-source review procedures.

SISMS consists of two linked databases: the Mass Public Shootings Event (MPSE) database and the Pre-Attack Communications and Behaviors of Mass Public Shooters (PACB-MPS) database (Table 1). The MPSE database captures incident-level characteristics related to the shooting event itself, including variables associated with location, victimization, weapons, casualties, and incident resolution. The PACB-MPS database captures perpetrator-level information related to demographics, grievances, concerning behaviors, planning and preparation, leakage, interpersonal stressors, behavioral dysregulation, substance use, and other pre-attack indicators associated with pathways to intended violence. The two databases are linked through shared event identification (EID) and perpetrator identification (PID) variables, allowing the datasets to be mapped and merged for both event-level and perpetrator-level analyses.

**Table 1:** Overview of the SISMS Dataset Structure

**Database**	**Unit of Analysis**	**Variables**	**Description**
MPSE	Incident/Event	23	Event-level characteristics related to the shooting incident
PACB-MPS	Perpetrator	271	Perpetrator-level characteristics related to pre-attack behaviors and background factors

## Data Collection and Coding Procedures

**Fig. 1:**
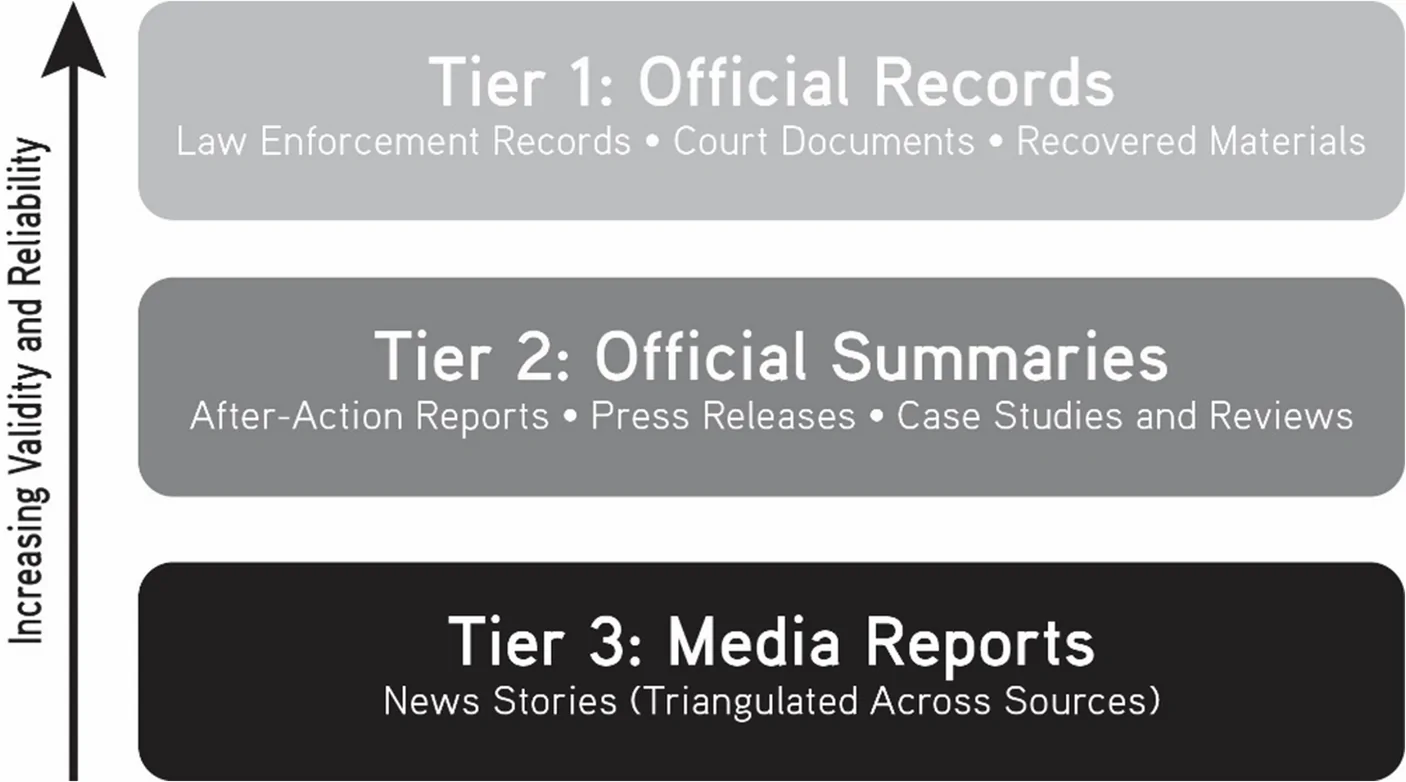
Three-Tier Framework for Open-Source Data Collection and Verification in SISMS

Data included in SISMS were collected using a structured three-tier framework for open-source information gathering (see Fig. 1). Tier 1 sources consisted of official records generated through investigative, judicial, or governmental processes, including law enforcement reports, court records, search warrants, interview transcripts, and perpetrator-generated materials obtained through official channels. Tier 2 sources included official summary or interpretive materials, such as press releases, after-action reports, and governmental case studies. Tier 3 sources consisted of media reporting and other publicly available open-source materials used to supplement and triangulate information across cases. Consistent with prior recommendations related to open-source research (Chermak et al., 2025), source collection prioritized official records whenever available.

To support the development of the dataset, extensive public records requests were submitted to law enforcement agencies, courts, and other governmental entities associated with the included incidents. More than 280 requests were filed seeking investigative reports, court materials, interview records, and related official documentation. Since the availability and completeness of records varied substantially across cases, source collection incorporated multiple forms of open-source material, including searches across 29 search engines and media platforms, to maximize data completeness and verification. When media sources were used, information was triangulated across at least three independent and credible outlets before coding decisions were made. The dataset was constructed from more than 154,000 pages of source materials, the majority of which (> 93% by page count) were derived from official records.

Coding procedures were guided by a standardized codebook developed from prior empirical and theoretical literature related to mass shootings, targeted violence, and pathways to intended violence. Trained research assistants coded cases using structured coding forms designed to document both coded values and the supporting source materials used to justify coding decisions. Following initial coding, all cases underwent a multi-stage verification process in which project investigators independently reviewed coding decisions against the original source documentation. Discrepancies or contested coding decisions were resolved through investigator review and adjudication procedures designed to promote consistency and transparency across cases. Since coding relied on iterative verification and adjudication procedures conducted by project investigators, formal inter-rater reliability statistics were not calculated during the initial coding phase.

SISMS utilizes a presence-based coding framework intended to prioritize confirmatory evidence and reduce assumptions related to missing or incomplete information. Under this approach, variables were coded as present only when sufficient evidence existed to support confirmation within the available source materials. Variables were coded as absent only when the reviewed materials provided sufficient information to reasonably conclude that the characteristic or behavior was not present. In cases where available records were insufficient to determine presence or absence, variables were treated as missing rather than inferred. This approach was intended to improve transparency and reduce the likelihood of overinterpreting incomplete open-source information.

## Research Applications

The SISMS dataset is designed to support a broad range of research applications related to mass public shootings, targeted violence, and violence prevention. The behavioral depth of the PACB-MPS database allows researchers to examine patterns related to grievances, concerning behaviors, leakage, planning and preparation, interpersonal stressors, behavioral dysregulation, and other pre-attack indicators associated with the Pathway to Intended Violence framework, which conceptualizes targeted violence as a process involving the progression from grievance to attack-related behaviors (Calhoun & Weston, 2003). The dataset also supports analyses examining the accumulation and co-occurrence of behavioral and psychosocial factors across perpetrators, as well as the identification of patterns that may inform prevention, intervention, and threat assessment efforts.

SISMS also allows researchers to examine relationships between pre-attack behaviors and incident-level characteristics contained within the MPSE database. The shared EID and PID variables further support integration with external demographic, geographic, policy, and contextual datasets, creating opportunities for multilevel and comparative research. In turn, findings derived from these analyses may help inform evidence-based prevention, intervention, and threat assessment practices relevant to criminal justice professionals, educators, behavioral threat assessment practitioners, policymakers, and other violence prevention stakeholders. As future phases of the dataset are completed, SISMS is expected to support comparisons between incidents with and without documented pre-attack warning behaviors or leakage.

## Strengths and Limitations

SISMS possesses several strengths that distinguish it from many existing open-source mass shooting datasets. Most notably, the dataset prioritizes official records and investigative materials as the foundation for data collection, reducing exclusive reliance on media reporting and allowing for substantially greater behavioral depth across cases. The linked structure of the MPSE and PACB-MPS databases further supports multilevel analyses examining relationships between perpetrator characteristics, pre-attack behaviors, and incident-level outcomes. In addition, the use of structured verification procedures and a presence-based coding framework was intended to improve transparency and reduce assumptions associated with incomplete or inconsistent open-source information.

At the same time, several limitations warrant consideration. As with all open-source datasets, the completeness and quality of available information varied across incidents, resulting in differing levels of detail and missingness across variables and cases. Official records also were not uniformly available, and some agencies declined or restricted records requests. Consequently, the absence of documented behaviors or characteristics should not necessarily be interpreted as evidence that such factors were not present. Since Phase 1 cases were selected based on the presence of documented warning behaviors or leakage (Greene-Colozzi, 2022), prevalence estimates derived from the current release should not be interpreted as representative base rates for the broader population of mass public shootings. Additional cases currently undergoing coding and verification procedures are expected to expand the analytic utility and comparative scope of the dataset in future releases.

## Conclusion

As research on mass public shootings continues to evolve, transparent and behaviorally rich data resources remain critical for advancing cumulative empirical knowledge. This short report introduced SISMS, a multilevel dataset designed to support research related to mass public shootings, pre-attack behaviors, and pathways to intended violence in the United States. By integrating linked event-level and perpetrator-level data derived primarily from official records, SISMS was developed to support both empirical inquiry and evidence-informed prevention efforts. As future phases of the project are completed, SISMS is expected to continue supporting research, prevention, and violence intervention efforts across a range of disciplines and professional contexts.

## Data Availability

The SISMS methodology guide and codebooks are publicly available through the SISMS project website (https://rockinst.org/sisms) hosted by the Regional Gun Violence Research Consortium. Researchers interested in obtaining the dataset may submit a data request form through the project website, subject to applicable data access and use procedures.
